# Systems Silver Iodide-Sodium Iodide and Silver Iodide-Potassium Iodide^1^

**DOI:** 10.6028/jres.064A.041

**Published:** 1960-10-01

**Authors:** G. Burley, H. E. Kissinger

## Abstract

The phase relations for the systems AgI-NaI and AgI-KI have been determined for the temperature range from room temperature to 685° C, using differential thermal analysis techniques. The AgI-NaI system has a eutectic at 50 mole percent NaI and 384° C. The AgI-KI system has eutectics at 20.8 and 28.5 mole percent KI and 254° C and 244° C, respectively. A compound of formula KAg_3_I_4_ is formed with a congruent melting point of 268° C.

## 1. Introduction

During the last decade, silver iodide has been used extensively in weather modification attempts. Because of the crystallographic similarity, silver iodide can serve as an epitaxial nucleation site for the growth of ice crystals. This greatly decreases the supercooling tendency of water vapor in the atmosphere.

One technique of generating a smoke of this substance consists of dissolving a mixture of AgI and an alkali iodide in acetone and feeding the resulting solution into the nozzle of a burner [1].^2^ Under normal operating conditions this gives particles ranging from 10^−2^ to 10^−3^ *μ* in diameter, and produces about 10^12^ nuclei per second.

Since both AgI and either NaI or KI are in the original solution and the solvent is presumably evaporated in the burner, the solid state relations of these constituent compounds are of interest. Mason [2] has reported that the product resulting from the combination of AgI and KI had little relation to either of the original compounds. This investigation of the phase relationships in the systems AgI-NaI and AgI-KI was undertaken in order to obtain information on equilibrium conditions in these systems.

## 2. Apparatus

A differential thermal analysis (DTA) unit was used to obtain most of the data. The apparatus has been described previously [3]. Specimen holders made of 5 mil platinum sheet in the form of tubular cups 4 cm in depth and 1 cm in diameter were used. The bottom of each container included a closed reentrant central tube, extending one-half the length of the cup, which fitted over the thermocouple junction. This arrangement served to contain the molten sample and to protect the thermocouples. Thermocouples were made of 15 mil Pt-Pt 10 percent Rh wire. The reference material was aluminum oxide.

The heating rate of the furnace was generally 5° C/min. The rate was reduced to 2° C/min to resolve complex peak shapes. The thermocouples were calibrated by using the quartz inversion at 573° C as an external standard. In addition, AgI has a reversible transformation at 146° C. This transformation produces a well defined thermal arrest which was used as an internal standard.

Near the eutectics and near the end member compositions, melting was accompanied by the characteristic endothermic DTA deflection. For intermediate compositions, the beginning and end of melting was indicated by a change in the slope of the base line.

A high-temperature powder X-ray goniometer [4] was used to give an approximate indication of melting points. Since the pattern decreases in intensity as the temperature is increased, the exact temperature of disappearance is difficult to determine by this method. For this reason all results reported here are those obtained with the DTA apparatus. The X-ray method was useful, however, in checking for possible phase changes having negligible heat effects.

## 3. Materials

The materials used were reagent grade chemicals. No special purification procedures were used. Chemical analysis supplied by the manufacturer showed a maximum of 0.01 percent Cl^−^ and 0.0003 percent ${\text{IO}}_{3}^{-}$ for the KI and 0.02 percent Cl^−^ and 0.001 percent ${\text{IO}}_{3}^{-}$ for the NaI. In order to minimize absorption of moisture from the atmosphere all transfers from reagent to weighing bottles were done in a drybox through which a stream of dry air was passed. The components were thoroughly mixed and then immediately used as starting materials for the DTA. In a number of cases, the thoroughly mixed reactants were melted, ground, mixed, and remelted before using them for the DTA. The results appeared to be identical with those obtained using mechanical mixtures, and it must be assumed that diffusion rates are high enough to assure rapid mixing and equilibrium.

## 4. Results

**Figure 1:**
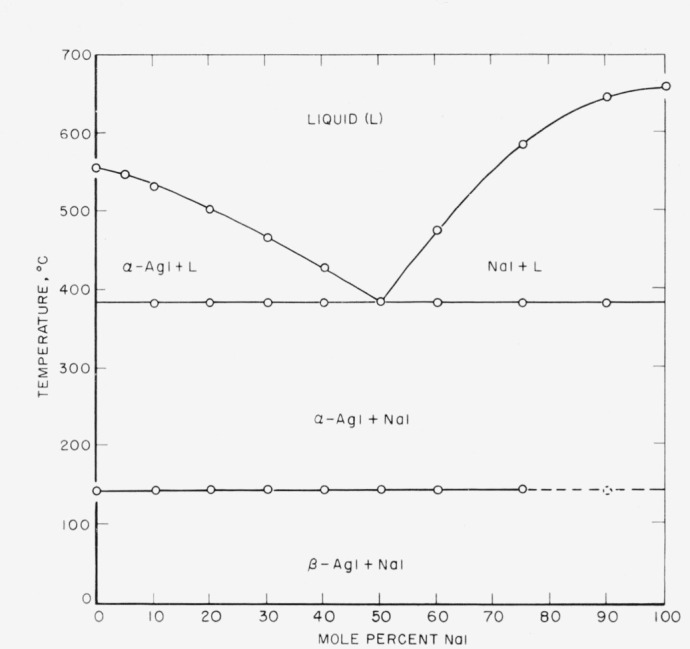
Phase diagram of the system AgI-NaI.

**Figure 2:**
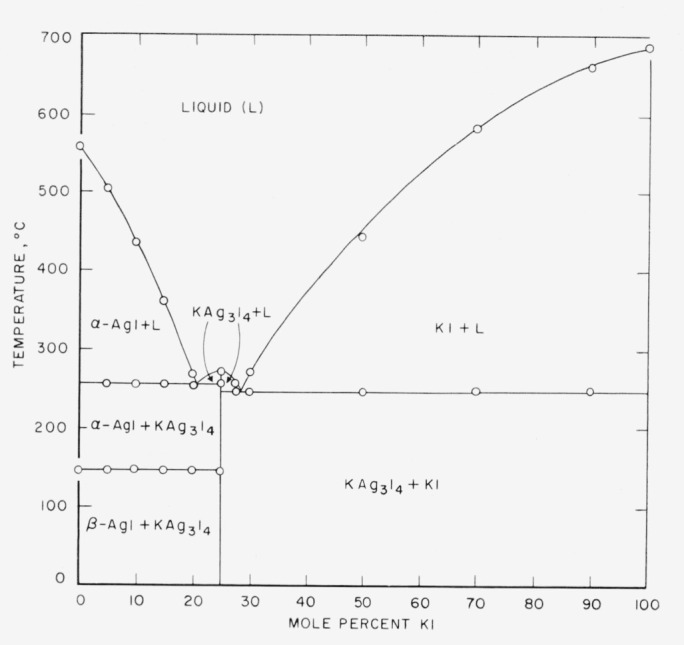
Phase diagram of the system AgI-KI.

The phase diagram of the system AgI-NaI is given in figure 1 and that of the system AgI-KI in figure 2.

Silver iodide has two low-temperature modifications [5]. These are a face-centered cubic sphalerite type (*γ*) structure and a hexagonal wurtzite type (*β*) structure. Only the latter appears to be stable [6]. At 146° C these change reversibly to a body- centered cubic type (*α*) structure which persists to the melting point at 555° C [7].

Sodium iodide has the face-centered cubic sodium chloride type structure. No phase changes occur to the melting point at 662° C [7]. Potassium iodide has the same structure type as sodium iodide and no phase changes. Its melting point is 685° [7].

The AgI-NaI system has a eutectic at 50 mole percent NaI and a temperature of 384° C. No solid solution region was observed. No compound formation occurs. The AgI transformation at 146° C apparently persists across the entire field, although it is difficult to observe at low AgI concentration.

The AgI-KI binary phase diagram shows two eutectics and formation of a compound. The eutectics are at 20.8 mole percent KI and 254° C and at 28.5 mole percent KI and 244° C. A 3AgI·KI compound is formed with a congruent melting point at 268° C.

## 5. Accuracy

The reproducibility and accuracy of the composition of the starting materials is estimated at ±0.5 mole percent because of the hygroscopicity of the alkali iodides. The temperature of a transformation appears to be reproducible to ±1° C in the DTA apparatus. In many cases solidification involved some supercooling, so that the melting temperatures were judged more reliable. The temperature of a eutectic, when far from the eutectic composition, is subject to a somewhat greater error. In general, the cumulative errors of composition, temperature measurement, and possible loss of iodine are estimated to amount to no more than ± 1 mole percent for each measured composition and ±2° C lor each measured temperature. Interpolated values are subject to the additional errors inherent in this procedure.

## Footnotes

2 Figures in brackets indicate the literature references at the end of this paper.
